# Supplementary material for: Development of a computer-aided design software for the quantitative evaluation of aesthetic damage
Source: PLoS One. 2019 Dec 18;14(12):e0226322. doi: 10.1371/journal.pone.0226322 (PMC6919621; doi:10.1371/journal.pone.0226322)
Supplement: S1 Code site — 1. (ZIP) [file pone.0226322.s001.zip › [EESC_jr][Projeto]Relatorio_07_21/reports/15/2017.04.04/12:17:09/report.pdf]

Preâmbulo

Na data de 09/03/2017 no horário 16:30, o (a) Perito(a) Ms. Nicole Prata Damascena, Cirurgiã-dentista, designado(a)para proceder ao exame pericial solicitado em: Clamarcia Santos, do sexo Feminino, nascido(a) em 31/08/1967, ocupação Auxiliar de limpeza, portador(a) do RG 878738 , cujo endereço Estância/SE, passa a expor a avaliação de dano estético corporal.

Exame objetivo da lesão

A lesão ora objeto de análise teve como natureza Acidente de trânsito ocorrido em 26/10/2015. O prejuízo estético consiste em lesão cicatricial consolidada na região: Rosto, Mento, Lábios, Pescoço no lado inferior, esquerdo, direito.

Trata-se de cicatriz Retrátil de tamanho/frequência Múltipla: Retilínea de orientação Oblíqua de pigmentação Hipocrômica medindo 0 centímetro(s) na horizontal, 2 centímetros na vertical e 0 milímetros de Altura. De contornos Regulares. Perceptivel em 100 centímetros de distância nas relações íntimas. Perceptivel em 4 metros nas relações sociais. A lesão tem repercução na vida diária afetiva, familiar, social conforme fotos anteriores e atuais..

Conclui-se que classificada segundo os parâmetros de dano estético AIPE - Análise do Impacto do Prejuízo Estético (escala de seis níveis e pontuação entre 0 e 50 pontos), como sendo dano estético relativo à área 1, a lesão é caracterizada por 7 pontos e grau de prejuízo estético: Leve.

Escore final: 7;
Grau de prejuízo: Leve.

Aracaju/SE, 09/03/2017

____

Ms. Nicole Prata Damascena
